# Variability of peak speed and sprinting actions during the same small-sided games: within- and between-player variations inspected over four consecutive weeks

**DOI:** 10.5114/biolsport.2023.124846

**Published:** 2023-02-03

**Authors:** Piotr Makar, Ana Filipa Silva, Adam Kawczyński, Zeki Akyildiz, Mehmet Yıldız, Gibson Praça, Filipe Manuel Clemente

**Affiliations:** 1 Gdańsk University of Physical Education and Sport, Poland; 2 Escola Superior Desporto e Lazer, Instituto Politécnico de Viana do Castelo, Rua Escola Industrial e Comercial de Nun’Álvares, 4900-347 Viana do Castelo, Portugal; 3 Research Center in Sports Performance, Recreation, Innovation and Technology (SPRINT), 4960-320 Melgaço, Portugal; 4 The Research Centre in Sports Sciences, Health Sciences and Human Development (CIDESD), 5001-801 Vila Real, Portugal; 5 Faculty of Sport Sciences, Gazi University, 06560 Ankara, Turkey; 6 Afyon Kocatepe University Sports Science Faculty, Turkey; 7 Sports Department, Universidade Federal de Minas Gerais, Belo Horizonte, Brazil; 8 Instituto de Telecomunicações, Delegação da Covilhã, Lisboa 1049-001, Portugal

**Keywords:** Football, Exercise testing, Physical assessment, Training monitoring, External load

## Abstract

This study aimed to analyze within- and between-player variations of peak speed and sprinting actions occurring in small-sided games (SSGs: 1 v 1 and 5 v 5). The study followed a cohort study design. Twenty male youth soccer players (age: 17 years old) from the same team were observed over four consecutive weeks. Each week, the players participated in two sessions (day one and day two) during which SSGs were applied. The 1 vs. 1 format was employed with four repetitions of thirty seconds interspaced with two-minute rest intervals, while the 5 vs. 5 format with four repetitions of four minutes and two-minute rest intervals between them. The players were monitored during all training sessions with the Polar Team Pro. The peak speed attained in each game, and the number of sprints were extracted as the primary outcomes. The between-player variability revealed a lower coefficient of variations for peak speed in the 1 vs. 1 (13.9%) and 5 vs. 5 (10.9%) formats than for sprints (1 v 1: 64.7%; 5 v 5: 65.5%). Considering the within-player variability, it was observed that sprints were more variable (1 vs. 1: 62.1%; 5 v 5: 65.7%) than peak speed (1 vs. 1: 16.4%; 5 v 5: 14.0%). The between-session analysis revealed that during week 1 (day 1), peak speed was significantly higher than during week 3 (day 1) in the 1 vs. 1 format (+3.0 km/h; p = 0.031; d = 1.296). Moreover, peak speed during week 3 (day 2) was considerably lower than during week 1 (–5.9 km/h; p < 0.001; d = 1.686) and week 2 (–5.0 km/h; p = 0.001; d = 1.639). The between-session analysis showed no significant differences in the sprint between the sessions on day 1 (p > 0.05). However, on day two, the sprint was substantially higher during week one than during week four in the 5 vs. 5 format (+5.40 n; p = 0.002; d = 2.571). In conclusion, this study revealed that peak speed presents lower within- and between-player variability than the number of sprints. Considering these two measures, there are no considerable variations between the weeks. Coaches should consider identifying strategies to stabilize the stimulus regarding the number of sprints if this represents one of the targets for employing SSGs.

## INTRODUCTION

Small-sided games (SSGs) are widely used in soccer training [1]. Among the main reasons they are such a common training tool is the possibility to integrate technical, tactical, and physical stimuli [2] and the constraints manipulation that allows directing adaptation towards training goals [3, 4]. However, as in all game-based training scenarios, small-sided games are subject to intrinsic variability from session to session (between-session variability) and from bout to bout (within-session variability), which might be seen as a threat to this training tool [5]. Although variations in match-related variables, due to the unpredictable nature of match-actions, are extensively reported in the literature [6], enormous variations in players’ responses during training sessions with small-sided games might bias training planning. For example, a coach’s ability to choose a specific format [7] to nurture a particular physical attribute (e.g., accelerations) might be impaired if the same SSG allows players to experience five accelerations per minute in one training session but only one action per minute in the following session. For this reason, it is worth investigating locomotor responses’ within- and between-variability when adopting SSGs in soccer training.

Previous studies showed contradictory results on this topic. On the one hand, a recent systematic review indicated that high-intensity locomotor demands presented high between- and within-session variability [8]. However, total distance and distances at low speeds showed small-to-moderate within-session variability. On the other hand, another study revealed that both GPS and accelerometer-based variables showed excellent reliability (low variability) in the between-session analysis [9]. The differences in the study designs and the control of intervening variables might explain the dissimilarities in the results. Further research is required due to the need for more agreement in the literature on this topic.

Although SSGs might present inherent variability, the possibility of manipulating task constraints might affect the variability of responses across multiple trials [10]. For example, it has been shown that physiological responses were less variable in 4 vs. 3 than in the 3-a-side SSG [11]. On the other hand, another study concluded that increasing a format (from 2-a-side to 6-a-side) did not affect the variability of physical responses [12]. However, continuous SSGs showed lower variability when compared to interval formats. Similarly, there were no differences in reliability levels between games with and without the offside rule [9], although the non-offside condition is likely to increase spatial exploration [13]. These variations are not restricted to locomotor responses, as they are also evident in technical [14] and tactical [11] responses. Besides the inconclusive evidence of the influence of the SSG format on the variability of locomotor responses, different SSGs can elicit different responses from players. For example, larger formats are usually associated with larger areas, allowing players to cover distances at higher speeds than smaller formats [15, 16]. This indicates that manipulating the SSG format makes it possible to induce different adaptations from players. Consequently, by investigating the variability of responses of different SSGs – such as 1 vs. 1 and 5 vs. 5 – coaches can be provided with valuable information on which configurations might present more stable responses over time.

While variability might play an important role in youth soccer regarding tactical development [17], it might negatively affect the quality of the training prescription regarding locomotor responses. Previous studies showed that within the same session, players positively change their tactical responses [18]. Consequently, it is expected that within-session variability can be present due to players’ adaptability to the task. Additionally, previous studies reported that SSGs could provide adequate stimuli to develop aerobic skills [19, 20]. Therefore, owing to physical improvements over time, between-session variability can also be expected, irrespective of the training format. Finally, as players tend to have more options to act and explore larger areas in larger formats, the format may affect variability and lower locomotor variations in smaller formats. Based on this rationale, the aims of the study are twofold. Firstly, to investigate the within- and between variability in peak speed and sprinting actions during small-sided soccer games, and secondly, to compare variability between 1 vs. 1 and 5 vs.5. The formats were selected because the 1 vs. 1 represents the minimum format occurring in SSG (a duel) in which individual participation is exacerbated. The 5 vs. 5 was selected considering that it is one of the most typical formats employed in SSGs representing medium sided-game typically used for imposing a high metabolic effort while having the opportunities to explore the main tactical and collective principles of play and in which the individual participation is regulated for the collective dynamics.

## MATERIALS AND METHODS

### Study design

The current research followed a repeated measures design in which the same participants took part in each condition of the independent variable (format of SSGs). Half of the participants were enrolled in performing the 1 vs. 1 format two days (day one and day two) a week over four consecutive weeks, while the other half completed the 5 vs. 5 format two days (day one and day two) a week over four consecutive days.

### Setting and context

**FIG. 1:**
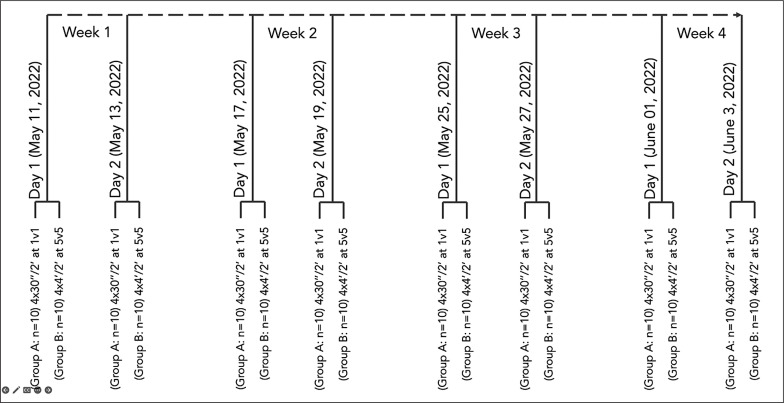
Timeline of the study.

Figure 1 presents the timeline of the study. The observation period started on May 11, 2022, and ended on June 3, 2022. The sessions began at the same time (5 pm) and were preceded by 24-h rest. The sessions always occurred at the same pitch (synthetic turf). The environmental conditions were as follows: May 11, temperature 23º and relative humidity 65%; May 13, temperature 21º and relative humidity 57%; May 17, temperature 26º and relative humidity 63%; May 19, temperature 19º and relative humidity 67%; May 25, temperature 17º and relative humidity 65%; May 27, temperature 16º and relative humidity 62%; June 01, temperature 28º and relative humidity 43%; and June 03, temperature 14º and relative humidity 68%.

### Participants

A convenience sampling method was employed. The sample size was calculated a priori in G*power (version 3.1.9.6.). For power = 0.80, effect size = 0.5 (medium), the recommended total sample was 21. The participant recruitment was performed among 23 players of the same team. The inclusion criteria for the study were as follows: (i) outfield players; (ii) players who participated in all training sessions on the days of data collection (8 training sessions over four consecutive weeks); (iii) players who did not take any drugs or energy drinks, or who did not change any of the daily dietary routines. From an initial group of twenty-three players, twenty male youth soccer players were selected (age: 17 years old; 7.5 ± 1.5 years of experience; 176 ± 5.3 centimeters of stature; 67.4 ± 4.9 kilograms of body mass; final velocity at 30–15 Intermittent fitness test: 19.6 ± 2.1 km/h).

All of them volunteered to participate in the study. Simple randomization was performed to allocate the participants into two groups: a group of the 1 v 1 format (n = 10; enrolled in 8 sessions of the 1 v 1 format) and a group of the 5 v 5 format (n = 10; enrolled in 8 sessions of the 5 v 5 format). The participants and their legal guardians were introduced to the study design and informed about the risks and benefits of participating. They were also reminded of their right to withdraw from the research without penalty. Once the participants agreed to participate in the study, the guardians signed a consent form. The study followed the ethical standards for medical research involving human subjects described in the Declaration of Helsinki.

### Small-sided games

The SSGs were preceded by the FIFA11+ warm-up programme [21]. The 1 vs. 1 and 5 vs. 5 formats was employed over eight sessions (two days a week over four consecutive weeks). One group of players (n = 10) performed only the 1 vs. 1 format, while the other group (n = 10) only used the 5 vs. 5 format. The characteristics of the 1 vs. 1 and 5 vs. 5 formats are presented in Table 1. The players were divided into teams based on the perception level of quality defined by the coach. The same players always had the same opponents and teammates (in the case of 5 v 5). [21]No verbal encouragement was given. The players were not provided with a strategic definition.

**TABLE 1:** Characteristics of the small-sided games.

	1 v 1	5 v 5
Scoring method	A small goal (2 × 2 m) without goalkeeper	A small goal (2 × 2 m) without goalkeeper
Pitch size	15 × 10 m	40 × 30 m
Length: width ratio	1.5	1.3
Area per player	75 m^2^	120 m^2^
Repetitions per session	4	4
Time per repetition	30 seconds	4 minutes
Rest between repetitions	2 minutes	2 minutes
Mode of rest	Passive	Passive
Verbal encouragement	No	No
Specific rules	No offside rule; Ball reposition with the foot	No offside rule; Ball reposition with the foot

### Monitoring peak speed and number of sprints

The players were monitored with the Polar Team Pro Global Positioning System (10 Hz, Polar, Finland). The devices had been previously tested and were found to have a good validity and reliability level to measure distances covered at different thresholds [22], particularly in determining peak speed [23]. The Polar Team Pro sensors were positioned in the center of each player’s chest and fixed with a band. The players always had the same sensor and band to avoid inter-unit variability. For each repetition, the peak speed detected and the number of sprints performed (running action performed at or above 25 km/h) was collected. For each session (day), the highest peak speed of all repetitions and the average number of sprints across repetitions were registered. These were the main outcomes used for further data treatment.

### Statistical procedures

The descriptive statistics is presented in a form of mean and standard deviation. The coefficient of variation (expressed as percentage) was also used to determine the between-session variability (heterogeneity of responses between the players) and the within-player variability (heterogeneity of responses of the same player across repeated measures). Normality of the data was checked using the Shapiro-wilk and was confirmed for different days and formats, taking into consideration both main outcomes (p > 0.05). Similarly, the Levene’s test also confirmed homogeneity of the sample (p > 0.05). Based on these assumptions, a mixed ANOVA (time*format of SSGs) was executed to analyze the variations of the main outcomes between the observation days. Partial eta squared () was used as the effect size for the mixed ANOVA. The Bonferroni test was used as post-hoc test. Additionally, the standardized effect size of Cohen (pooled standard deviation method) was executed to estimate the effect size of pairwise comparisons. The statistical procedures were executed in the SPSS software (version 28.0.0.0, IBM, Chicago, USA) for a p < 0.05.

## RESULTS

Table 2 presents the between- and within-player variability (expressed as coefficient of variation) for the 1 vs. 1 and 5 vs. 5 formats performed over a 4-week observation. The between-player variability (i.e., variability within the format and session) revealed a lower coefficient of variations for peak speed in the 1 vs. 1 (13.9%) and 5 vs. 5 (10.9%) formats than for sprints (1 vs. 1: 64.7%; 5 v 5: 65.5%). Considering the within-player variability (i.e., variability of the same player across different weeks), it was observed that sprints were more variable (1 vs. 1: 62.1%; 5 v 5: 65.7%) than peak speed (1 vs. 1: 16.4%; 5 v 5: 14.0%).

**TABLE 2:** Between- and within-player variability for peak speed and sprinting count in the 1 v 1 and 5 v 5 formats.

Outcome	Format	Overall (Mean ± SD)	Overall BPV (CV%)	Overall WPV (CV%)	Day 1 (Mean ± SD)	Day 1 BPV (CV%)	Day 1 WPV (CV%)	Day 2 (Mean ± SD)	Day 2 BPV (CV%)	Day 2 WPV (CV%)
Peak Speed (km/h)	1 v 1	21.1 ± 3.5	13.9	16.4	20.6 ± 3.1	13.2	15.1	21.7 ± 3.8	14.6	15.9
Peak Speed (km/h)	5 v 5	21.2 ± 3.1	10.9	14.0	20.2 ± 2.5	11.8	10.8	22.2 ± 3.3	10.0	15.0
Sprint (n)	1 v 1	5.4 ± 3.7	64.7	62.1	5.2 ± 2.8	52.9	47.7	5.7 ± 4.5	76.5	73.2
Sprint (n)	5 v 5	5.3 ± 3.8	65.5	65.7	4.6 ± 3.8	76.5	66.5	6.1 ± 3.7	54.2	57.8

BPV: between-player variability (represents the average of coefficient of variation within the format and session); WPV: within-player variability (represents the average of coefficient of variation of each player across the different days analyzed); SD: standard deviation

**FIG. 2:**
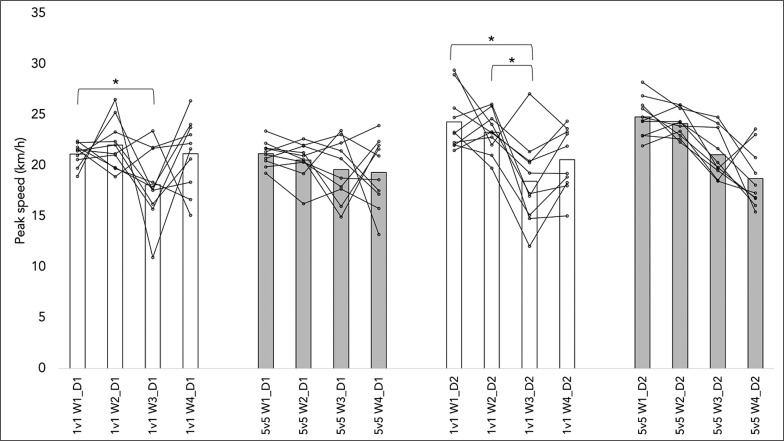
Within-player variability of peak speed across different formats and sessions analyzed. Legend: W: week; D: day; *: significant difference (p < 0.05) in the within-format comparison.

Figure 2 presents the within-player variability of the peak speed over the observation period. The Mixed ANOVA (time*format) revealed no significant influence on the peak speed of day one (F = 1.463; p = 0.246; = 0.075). However, there was a considerable impact on the peak speed of day two (F = 3.276; p = 0.028; = 0.154). There were no significant differences in the peak speed of day one between the 1 vs. 1 and 5 vs. 5 formats (p = 0.385) and day two (p = 0.450). The between-session analysis revealed that week 1 (day 1) had a noticeably higher peak speed than week 3 (day 1) in the 1 v 1 format (+3.0 km/h; p = 0.031; d = 1.296). Moreover, week 3 (day 2) had a significantly smaller peak speed than weeks one (–5.9 km/h; p < 0.001; d = 1.686) and two (–5.0 km/h; p = 0.001; d = 1.639).

**FIG. 3:**
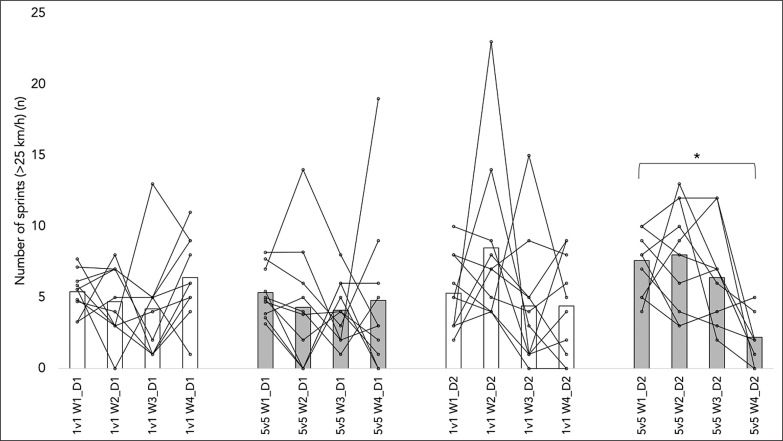
Within-player variability of a number of sprints across different formats and sessions analyzed. Legend: W: week; D: day; *: significant difference (p < 0.05) in the within-format comparison.

Figure 3 presents the within-player variability of the peak speed over the observation period. The Mixed ANOVA (time*format) revealed no significant influence on the number of sprints of day one (F = 0.272; p = 0.774; = 0.015) and day 2 (F = 1.497; p = 0.226; = 0.077). No substantial differences were found in the number of sprints of day one between the 1 vs. 1 and 5 vs. 5 formats (p = 0.556) and day two (p = 0.576). The between-session analysis revealed no considerable differences in the number of sprints between the sessions on day one (p > 0.05). However, on day two, the number of sprints was significantly higher during week one than during week 4 in the 5 v 5 format (+5.40 n; p = 0.002; d = 2.571).

## DISCUSSION

The current research revealed that the number of sprints presents higher between- and within-player variability. At the same time, the peak speed seems less variable in both play formats and across the repeated measures considered. Moreover, although considerable punctual differences were observed in the peak speed and the number of sprints between the weeks, most variations were insignificant. Finally, no substantial differences between the formats regarding the peak speed and the number of sprints were found.

Peak speed is one of the measures commonly inspected by sports scientists and coaches to identify a mechanical intensity level to which a player is exposed from a neuromuscular perspective [24, 25]. Peak speed is also a way to promote a mechanical stimulus to promote positive physical adaptation in soccer players [25, 26]. Since SSGs are common tasks coaches use to introduce a physical stimulus, it is important to understand how the between- and within-player variability can occur. The current research revealed that the peak speed presents the between-player variability around 11 and 14% (for the 5 v 5 and 1 v 1 formats, respectively) and the within-player variability around 14 and 16% (for the 5 v 5 and 1 v 1 formats, respectively). Although no comparative study has been reported, the variability of peak speed in small-sided games, the values registered are good since variability for high-speed running (as an example) usually varies between 45 and 146% [27]. Although the values attained were relatively low (~21 km/h), the variability within- and between players is not high, which is an exciting perspective for coaches. It is well-known that SSGs (notably smaller formats) do not allow a great velocity stimulus since fields are small and do not allow them to achieve high values of speed [28]. However, with the low between-player and within-player variability, it is more expectable for coaches to recognize the dose of stimulus that will be introduced. The fact that the 1 vs. 1 and 5 vs. 5 formats were used also weakened the hypothesis of high heterogeneity since a play is less structured than a bigger format, and players may play more similarly in a field.

The second main outcome analyzed in this research was the number of sprints. In this case, the variability was higher than the peak speed. The between-player variability fluctuated between 55 and 56% of the coefficient of variation, while within-player variability varied between 62 and 66%. Previous studies reported that sprints could vary between 50 and 140% [29]. Although GPS devices may be less accurate and generate greater errors in high-intensity demands, it is also important to mention that 10 Hz GPS is adequate to ensure the quality of the retrieved data [22]. One of the explanations for higher variability in the number of sprints can be the game dynamics which depends on contextual factors such as tactical behavior, match status, and opponent’s skills [30]. Therefore, higher variability is expected since the game dynamics may influence exposure to high-demanding running activities. This is also observed in regular official matches [31]. One of the indications that should be disclosed is that coaches must monitor locomotor demands in SSGs to avoid situations of under or over-stimulus. When using SSGs, it is important to monitor and compensate players with less stimulus with additional sprint exposure [24].

The current research also tested repeated measure variations for each of the formats of a play. Although some significant differences were observed, most pairwise comparisons did not reveal considerable changes. This may suggest that the pooled data may have hidden within-player variability, which should be considered an alert for coaches. Looking into the mean may not reveal how the load is floating for each player. Thus it is vital to apply strategies of locomotor demands and monitor to identify the impact of the on each player.

This study presents some limitations. Because a crossover design was not used in the study, it is impossible to identify the effects of testing the same players with other formats. Moreover, the data have been collected from the same team, which does not allow a profound generalization of the findings. Future research should consider the extension of the number of teams included and the identification of how other SSG constraints may play a role in variability. It is also recommended that future research may combine information from situational factors and tactical-technical variables while combining them with variations in locomotor responses. That will open the view for the characterization of the processes that may justify the variability.

However, despite the limitations, the study is one of the few centering research on the peak speed and the number of sprints variability in SSGs, which may help coaches to identify how the exposure of soccer players to this type of event can vary. It is recommended to monitor players during SSGs and to apply compensating strategies to stabilize the stimulus in players that require such attention.

## CONCLUSIONS

This study revealed that peak speed presents lower between- and within-player variability than the number of sprints in the 1 v 1 and 5 v 5 formats. Moreover, despite some differences in repeated measures over four weeks, most pairwise comparisons did not reveal significant changes in the exposure to the peak speed and the number of sprints. It is recommended that coaches should consider an individual monitoring process during SSGs and stay attentive to the variability of high-intensity demands, such as the number of sprints, to avoid under or over-stimulus for this important indicator.

## References

[1] Gonçalves L, Camões M, Lima R, Bezerra P, Nikolaidis PT, Rosemann T, et al. Characterization of external load in different types of exercise in professional soccer. Hum Mov. 2022; 23(1):89.95.

[2] Clemente FM, Afonso J, Sarmento H. Small-sided games: An umbrella review of systematic reviews and meta-analyses. Boullosa D, editor. PLoS One. 2021; 16(2):e0247067. 33577611 10.1371/journal.pone.0247067 PMC7880470

[3] Woods CT, McKeown I, O’Sullivan M, Robertson S, Davids K. Theory to Practice: Performance Preparation Models in Contemporary High-Level Sport Guided by an Ecological Dynamics Framework. Sports Med Open. 2020; 6(1):1–11. 32797290 10.1186/s40798-020-00268-5 PMC7427670

[4] Praça GM, Andrade AGP, Bredt S da GT, Moura FA, Moreira PED. Progression to the target vs. regular rules in Soccer small-sided Games. Sci Med Football. 2021; 6(1):66–71. 10.1080/24733938.2020.1869811 35236221

[5] Clemente F. The Threats of Small-Sided Soccer Games: A Discussion About Their Differences With the Match External Load Demands and Their Variability Levels. Strength Cond J. 2019; 42(3):100–105.

[6] Akyildiz Z, Nobari H, González-Fernández FTT, Praça GM, Sarmento H, Guler AH, et al. Variations in the physical demands and technical performance of professional soccer teams over three consecutive seasons. Sci Reports. 2022; 12(1):1–24. 10.1038/s41598-022-06365-7 PMC8844418 35165313

[7] Li Z, Mao L, Krustrup P, Randers MB. Internal and external load during 8 v 8, 5 v 5 and 3 v 3 in Chinese elite youth male football players. Biol Sport. 2022; 39(4):1065–71. 36247968 10.5114/biolsport.2022.113292 PMC9536380

[8] Clemente FM, Aquino R, Praça GM, Rico-González M, Oliveira R, Silva AF, et al. Variability of internal and external loads and technical / tactical outcomes during small-sided soccer games: a systematic review. Biol Sport. 2022; 39(3):647–72. 35959343 10.5114/biolsport.2022.107016 PMC9331334

[9] Custódio IJ de O, Praça GM, Paula LV de, Bredt S da GT, Nakamura FY, Chagas MH. Intersession reliability of GPS-based and accelerometer-based physical variables in small-sided games with and without the offside rule. Proc Inst Mech Eng P J Sport Eng Technol. 2022; 236(2):134–142.

[10] Riboli A, Esposito F, Coratella G. Technical and locomotor demands in elite soccer: manipulating area per player during small-sided games to replicate official match demands. Biol Sport. 2023; 40(3):639–47. 37398955 10.5114/biolsport.2023.118338 PMC10286612

[11] Bredt, Sarah; Praça, Gibson; Figueiredo, Lucas; Paula, Leandro; Silva, Patrick; Andrade, André; Chagas M. Reliability of physical, physiological and tactical measures in small-sided soccer games with numerical equality and numerical superiority. Rev Bras Cinean Hum. 2016; 18(5):602–10.

[12] Hill-Haas S, Coutts A, Rowsell G, Dawson B, Coutts A, Dawson B. Variability of acute physiological responses and performance profiles of youth soccer players in small-sided games. J Sci Med Sport. 2008; 11(5):487–90. 17825620 10.1016/j.jsams.2007.07.006

[13] Praça GM, Chagas MH, Bredt SGT, Andrade AGP, Custódio IJO, Rochael M. The influence of the offside rule on players’ positional dynamics in soccer small-sided games. Sci Med Football. 2021; 5(2):144–9. 10.1080/24733938.2020.1819559 35077330

[14] Clemente FM, Chen YS, Bezerra JP, Guiomar J, Lima R. Between-format differences and variability of technical actions during small-sided soccer games played by young players. Hum Mov. 2018; 19(5):114–20.

[15] Clemente FM, Praca GM, Teles Bredt S da G, van der Linden I CM, Serra-Olivares J. External load variations between medium- and large-sided soccer games: ball possession games vs regular games with small goals. J Hum Kinet. 2019; 70(1):191–8. 31915489 10.2478/hukin-2019-0031 PMC6942466

[16] Praça GM, Chagas MH, Bredt SDGT, Andrade AGP de. Small-Sided Soccer Games with Larger Relative Areas Result in Higher Physical and Physiological Responses: a Systematic and Meta-Analytical Review. J Hum Kinet. 2022; 81(1):163–76. 35291625 10.2478/hukin-2022-0013 PMC8884881

[17] Caso S, van der Kamp J. Variability and creativity in small-sided conditioned games among elite soccer players. Psychol Sport Exerc. 2020; 48:101645.

[18] Praça GM, Barbosa GF, Moreira PED, Sousa RBE, Bredt SGT, Chagas MH, et al. Changes in tactical behavior during small-sided and conditioned games performed within a training session. Rev Bras Cinean Hum. 2020; 22:1–11.

[19] Halouani J, Chtourou H, Gabbett T, Chaouachi A, Chamari K, Halouani, Jamel; Chtourou, Hamdi; Gabbett, Tim; Chaouachi, Anis; Chamari K. Small-sided games in team sports training: a brief review. J Strength Cond Res. 2014; 28(12):3594–618. 24918302 10.1519/JSC.0000000000000564

[20] Hammami A, Gabbett TJ, Slimani M, Bouhlel E. Does small-sided games training improve physical-fitness and specific skills for team sports? A systematic review with meta-analysis. J Sports Med Phys Fitness. 2018; 58(10):1446–55. 29072025 10.23736/S0022-4707.17.07420-5

[21] Bizzini M, Impellizzeri FM, Dvorak J, Bortolan L, Schena F, Modena R, et al. Physiological and performance responses to the “FIFA 11+” (part 1): is it an appropriate warm-up? J Sports Sci. 2013; 31(13):1481–90. 23855725 10.1080/02640414.2013.802922

[22] Akyildiz Z, Yildiz M, Clemente FM. The reliability and accuracy of Polar Team Pro GPS units. Proc Inst Mech Eng P J Sport Eng Technol. 2022; 236(2):83–89.

[23] Sagiroglu İ, Akyildiz Z, Yildiz M, Clemente FM. Validity and reliability of Polar Team Pro GPS units for assessing maximum sprint speed in soccer players. Proc Inst Mech Eng P J Sport Eng Technol. ahead-of-print.

[24] Buchheit M, Simpson BM, Hader K, Lacome M. Occurrences of near-to-maximal speed-running bouts in elite soccer: insights for training prescription and injury mitigation. Sci Med Football. 2021; 5(2):105–10. 10.1080/24733938.2020.1802058 35077328

[25] Thoseby B, D. Govus A, C. Clarke A, J. Middleton K, J. Dascombe B. Between-match variation of peak match running intensities in elite football. Biol Sport. 2022; 39(4):833–8. 36247963 10.5114/biolsport.2022.109456 PMC9536389

[26] Beato M, Drust B, Iacono A dello. Implementing High-speed Running and Sprinting Training in Professional Soccer. Int J Sports Med. 2021; 42(4):295–299. 33291180 10.1055/a-1302-7968

[27] Clemente FM, Aquino R, Praça G, Rico-González M, Oliveira R, Silva AF, et al. Variability of internal and external loads and technical/tactical outcomes during small-sided soccer games: a systematic review. Biol Sport. 2021; 3(39):647–72. 10.5114/biolsport.2022.107016 PMC9331334 35959343

[28] Castagna C, Francini L, Póvoas SCA, D’Ottavio S. Long-Sprint Abilities in Soccer: Ball Versus Running Drills. Int J Sports Physiol Perform. 2017; 12(9):1256–63. 28253025 10.1123/ijspp.2016-0565

[29] Hill-Haas S, Coutts A, Rowsell G, Dawson B, Coutts A, Dawson B, et al. Variability of acute physiological responses and performance profiles of youth soccer players in small-sided games. J Sci Med Sport. 2008; 11(5):487–90. 17825620 10.1016/j.jsams.2007.07.006

[30] Buchheit M, Simpson BM. Player-Tracking Technology: Half-Full or Half-Empty Glass? Int J Sports Physiol Perform. 2017; 12(Suppl 2):S2–35–S2–41. 10.1123/ijspp.2016-0499 27967285

[31] Carling C, Bradley P, McCall A, Dupont G. Match-to-match variability in high-speed running activity in a professional soccer team. J Sports Sci. 2016; 34(24):2215–23. 27144879 10.1080/02640414.2016.1176228

